# Co‐ideation and co‐design in co‐creation research: Reflections from the ‘Co‐Creating Safe Spaces’ project

**DOI:** 10.1111/hex.13785

**Published:** 2023-05-31

**Authors:** Scott J. Fitzpatrick, Heather Lamb, Erin Stewart, Amelia Gulliver, Alyssa R. Morse, Melanie Giugni, Michelle Banfield

**Affiliations:** ^1^ Centre for Mental Health Research, National Centre for Epidemiology and Population Health, ANU College of Health & Medicine The Australian National University Canberra Australia; ^2^ ACT Mental Health Consumer Network Canberra Australian Capital Territory Australia; ^3^ The ALIVE National Centre for Mental Health Research Translation Melbourne Victoria Australia

**Keywords:** co‐creation, co‐design, knowledge production, participatory research, patient and public involvement, safe spaces, suicide prevention

## Abstract

**Introduction:**

Numerous frameworks for defining and supporting co‐created research exist. The practicalities of designing and conducting co‐created research are clearly important, yet the utility of these frameworks and their operationalisation within local contexts and involving a diversity of stakeholders and interests are currently not well‐researched.

**Methods:**

Using an instrumental case study approach, we examined the utility of a published systematic framework designed to improve clarity about co‐creation as a concept and approach. The framework is explored based on the first two processes that correspond to our own work to date: co‐ideation and co‐design.

**Results:**

Our study showed that diverse stakeholders bring challenges regarding research priorities, methods, language and the distribution of power within co‐creation processes. Co‐creation activities were incremental, adaptable, responsive and made best use of established relationships, structures and collective leadership to meet the competing demands of funders and human research ethics committees, while ensuring the meaningful participation of multiple stakeholders.

**Conclusion:**

The findings highlight the iterative, fluid and deeply relational nature of co‐created research. Rather than seeking to categorise these processes, we argue that the social relations of research production that provide the structures within which all co‐created knowledge is generated are more important drivers of effective knowledge mobilisation and implementation. Thus, close attention to these social relations is needed in co‐created research.

**Patient or Public Contribution:**

People with lived experience of emotional distress and/or suicidal crisis, including academic researchers, service and peer workers, carers and advocates were involved in the co‐ideation and co‐design of this research. All authors identify as people with lived experience, from both academic and nonresearch backgrounds.

## 1 INTRODUCTION

### 1.1 Background

The inclusion of multiple stakeholders in health services research is recognised as highly beneficial to the design and delivery of services and programs that better respond to the needs of individuals and communities.^1^ Referring to collaborative research practices and related activities that support research engagement, translation and impact, co‐creation encourages the alignment of researcher aims and priorities with those of service‐users, practitioners and other end‐users.^2^, ^3^ Co‐creation is thus conceptualised as a dynamic, creative and collaborative approach to research that focuses on developing solutions to priority problems or issues.^4^ The outcomes of co‐creation range from individual to system‐level innovations. It can be used to improve clinical effectiveness, service user–provider relationships, and experiences of care.^4^, ^5^ In addition to healthcare quality improvements, co‐created research has been shown to benefit researchers, service‐users, family/caregivers and staff. For instance, it can ensure research questions and materials are relevant and culturally acceptable, as well as help build connections between health or social services, communities and service‐users.^3^, ^6^ For groups who have been traditionally marginalised and maligned by services, such as those who experience emotional distress and/or suicidal crises and their family/caregivers, research co‐creation can help bring about cultural change within services and research practice through improved communication, greater empathy and shared commitment.^4^, ^7^

Research targeting health services may involve a range of stakeholders including researchers, service providers, policy makers and funding bodies; yet, how such involvement is conceptualised and the rationales that support such approaches differ. Greenhalgh et al.^1^ identify three key reasons for engaging and collaborating with stakeholders in the design and delivery of services and programs. The first, described as emancipatory, is that service users, particularly those from marginalised groups, have the right to participate in research, policy making and service design that seeks to represent them and/or address their interests. The second is that involving multiple stakeholder perspectives lends itself to the more effective transfer of evidence into practice, increasing the efficiency and value of research.^8^ The third argument is that the co‐creation of knowledge by multiple stakeholders is a defining feature of contemporary science, and a means by which researchers and professional and public bodies can secure resources and increase the accountability and transparency of research. Despite this increased commitment to co‐created research, ongoing barriers include the lack of supportive institutional structures and evidence for its effectiveness.^3^, ^9^

### 1.2 A co‐creation framework

The collaborative processes by which new knowledge is generated are also highly variable and beset by definitional issues and the inconsistent use of terminology.^10^, ^11^ According to Pearce et al.,^11^ the concept of co‐creation has typically been defined in one of two ways: (i) as the collaborative generation of new knowledge, or (ii) as the planning, implementation and evaluation of new services and programs. They contend that a complementary framework that acknowledges and incorporates both aspects is key to the concept of co‐creation and its capacity to address problems of collaborative involvement and power that can result if there is no requirement for collaboration.^11^ This is particularly relevant in fields such as suicide prevention where the generation of new knowledge often occurs in parallel with the design, delivery and evaluation of new services and programs. The wide variability of co‐creation‐related terms such as co‐design, co‐production and co‐planning, moreover, suggests a range of distinct approaches rather than a similar approach being adapted across different contexts.^11^ Seeking to improve clarity about co‐creation as a concept and approach, Pearce et al.^11^ identify four primary categories of collaborative processes in the research literature: (1) co‐ideation, (2) co‐design, (3) co‐implementation and (4) co‐evaluation.

In this paper, using a case study approach, we seek to explore the utility of the framework by describing two of these categories in our current co‐creation research project: co‐ideation and co‐design. In doing so, we seek to document and reflect upon these processes and key challenges within a national multisite research project involving a team of academic researchers (including researchers with lived experience of suicidal crisis or distress), health and community service managers, peer workers and lived experience advocates. We have chosen to publish our learnings on these initial stages of the project both as a means of guiding our own reflective processes for the co‐implementation and co‐evaluation stages, and to provide timely testing and development of Pearce et al.'s^11^ framework. An anticipated second paper will address the remaining two processes: co‐implementation and co‐evaluation.

## 2 METHODS

To gain a broader understanding of co‐creation as a concept and process, and to examine the utility of Pearce et al.'s^11^ framework, we undertook an instrumental case study grounded in our own experience of designing and coordinating the Co‐Creating Safe Spaces project. The instrumental case study is well‐suited to the study of practical and policy issues in health service settings.^12^ Capturing salient information on ‘more explanatory “*how*”, “*what*” and “*why*” questions’ including those of an interpretative or critical nature, the case study allows researchers to generate in‐depth, multifaceted understanding of broad, complex questions in their real‐life contexts that are potentially transferable to other contexts.^13^

The Co‐Creating Safe Spaces project has been designed to embed co‐creation into all aspects and stages of research from conception through to dissemination. Safe spaces are peer‐led alternatives to hospital emergency departments. They are specifically co‐designed with people with lived experience of emotional distress and/or suicidal crisis and offer safe, accessible, recovery‐oriented support to people.^14^ The widespread development of safe spaces in Australia involved an extensive co‐design process involving health professionals and people with lived experiences of emotional distress and/or suicidal crisis. Findings from these activities served as the starting point for the development of safe space models funded by government and government‐funded organisations tasked with commissioning services that meet local needs.^15^ Key components of safe spaces are: (1) a trauma‐informed ‘no wrong door’ approach, (2) nonclinical support that meets the holistic needs of guests, (3) a compassionate and capable peer‐led workforce, (4) a safe and accessible location, (5) a warm and welcoming environment, (6) warm connections and appropriate and reliable supports and (7) shared governance and management.^15^

The development of safe and accessible nonclinical spaces where people can receive support from others who have survived their own experiences of suicidality marks a radical departure from mainstream medical approaches to suicide. Such change has the potential to challenge existing power structures. The inclusion and respect for lived experience voices in Co‐Creating Safe Spaces extends this challenge, and signals the importance of emancipatory research principles in the co‐creation process, including a commitment to health systems change and improving human experience.^4^ The benefits of a research approach that comprises and respects a significant element of lived experience, with nonacademic and nonresearch‐based co‐investigators, include a more equal relationship between researchers and research participants, and a strong commitment to improving service users' experiences.^16^ At the same time, this commitment to implementing culture change in health systems and advancing new ways of helping those in emotional distress and/or suicidal crisis is offset by the requirements of ongoing performance monitoring and evaluation within health policy settings, and the demand for rigorous research methods to assess process, impact and outcomes to secure ongoing funding.^11^, ^17^ In light of these challenges, we drew on a critical, reflective perspective to our case study that sought to take into account shared social meanings and processes, the wider institutional contexts that shaped co‐creation, and the importance of power‐knowledge relations.^13^

## 3 FINDINGS

### 3.1 Co‐ideation and early challenges

Co‐ideation, according to Pearce et al.,^11^ involves the use of collaborative dialogue to generate and share new and creative ideas in relation to meeting community needs, solving problems and improving service design and delivery. The shift toward the implementation of co‐created, peer‐led, nonclinical services marks a proactive response to the unmet needs of those experiencing emotional distress and/or suicidal crisis, who frequently report negative experiences of emergency department care.^18^ At the same time, concerns about the short‐termism of health services mean there is considerable pressure to determine the effectiveness of these services; particularly as genuine, viable and sustainable alternatives for people who might usually present to the emergency department or choose not to access help through an emergency department due to past negative experiences.

ACACIA: The ACT Consumer and Carer Mental Health Research Unit, Centre for Mental Health Research, at the Australian National University sought to address this challenge. Following an initial request from ACT (Australian Capital Territory) Health to contribute to an evaluation of the local safe space pilot under development, the opportunity arose to apply for competitive funding for multisite research to advance the evidence base for nonclinical alternatives to emergency departments and hospital‐based care for people experiencing emotional distress and/or suicidal crisis. These targeted research grants were intended to support research partnerships between researchers in suicide prevention, lived experience partners, and suicide prevention/postvention programs and services. This provided an ideal opportunity to pursue co‐creation methods.

Research funding cycles are not co‐creation friendly.^9^ There is typically insufficient time to build trusting relationships where power is truly shared to a necessary degree for effective co‐creation. This is especially challenging where the project involves multiple stakeholders across multiple sites. In the initial stages of the Co‐Creating Safe Spaces grant preparation, formation of the project team leveraged already established relationships and structures. The ACACIA Advisory Group, which comprises independent consumer and carer representatives, staff and consumer representatives from Territory consumer and carer peaks, and representatives from ACT Health played a central role in early co‐ideation activities. These relationships, together with the existing collaboration on the pilot ACT safe space evaluation via the ACT Safe Haven Steering Committee, established a solid basis for co‐ideation. At this stage, co‐ideation centred primarily on how best to embed evaluation within the safe space to ensure that sufficient evidence was generated from the pilot to commission safe space services in the longer term.

### 3.2 Multiple stakeholders and diverse views in co‐ideation

The importance of the research being integrated within the health services that deliver the safe space programs, together with the engagement and inclusion of lived experience voices and perspectives, requires multiple stakeholder engagement and input. The cultivation of an iterative, incremental and participatory approach, therefore, commenced early on, and was critical to the progression of the research and attendant processes of co‐ideation and co‐design that took place during the development of the funding proposal. The research study also sought to build a culture of equal and shared knowledge across all stakeholder groups. Members of safe space steering committees from three services and Roses in the Ocean, a leading Australian lived experience of suicide organisation, contributed to the shaping of research questions and the writing and reviewing of the funding proposal, as well as the approach to co‐creation. Researchers from four Australian universities were invited to participate and to contribute content and methodological expertise on areas related to suicide and aftercare responses, health services evaluation, and the translation and implementation of recommendations. Many of these academic researchers also identified as having a lived experience which informed their work. To complement these academic roles, two paid positions were created for lived experience researchers with a nonacademic background to join the project as co‐investigators with negotiable time commitment and an equal say in decision‐making. All paid roles within the project are identified lived experience research roles.

Such diversity of stakeholders brings with it a range of views and challenges. One prominent example was the causal relation between suicide and mental illness, which is contentious and posed several challenges. While people with psychiatric disorders are at increased risk of suicide, it was acknowledged that people can experience emotional distress and/or suicidal crisis in the absence of any underlying mental health condition. Some stakeholders within the project have firm mental health services and policy connections, whereas others position suicide prevention in a separate but parallel space. Differing views on these issues among stakeholders shaped the language that was used in the funding proposal, as well as discussions around how the effectiveness and acceptability of models of care, service locations, and their reach would be evaluated. An established implementation science framework, RE‐AIM^19^ was chosen to add critical structure to the project, but how this was described and operationalised to be consistent with the co‐designed, nonclinical safe spaces across multiple sites required purposeful attention. All services in the project were developed and implemented with involvement from the State or Territory Government mental health departments, so it was important to reflect connections with mental health in the proposal, but this was balanced with nonclinical terms such as ‘emotional distress’ to encompass the range of experiences targeted by the safe spaces.

### 3.3 Approaches to evaluation design

Diverse views on the importance of different outcomes and what constitutes rigorous research methods were also a distinguishing feature of the co‐ideation process. The project was layered to enable both an overarching understanding of the implementation of safe spaces, and individual site evaluations. The decision to deconstruct a large evaluation into smaller components enabled the team to retain a focus on the experiences of safe space guests (those who access the safe spaces for support) that was closely aligned with the aim to harness lived experience knowledge across the whole of the project. This was complemented with a system perspective using implementation science frameworks to assess the impact of safe spaces on different dimensions and outcomes related to the implementation of these models, emergency department presentations, costs and care pathways.

### 3.4 Co‐design values and principles

Co‐design of the Co‐Creating Safe Spaces research project was separate to the co‐design of the safe spaces themselves. The aims, methodology and methods of the project, however, were guided by the values and principles identified in the initial co‐design of the safe spaces. These include respect, inclusion, choice, transparency, safety, lived experience‐led and valuing each person's experience and expertise.^15^ Consideration of these values and principles, and, in particular, how to embed lived experience in developing co‐designed research methods in a transparent and inclusive manner while also providing flexibility, choice and ease‐of‐use was the major focus of the co‐design process. We commenced this process by holding a co‐design workshop that brought together the core group of researchers, key stakeholders and site partners including managers, team leaders and peer workers. The aims of this workshop were to establish shared values and principles for working together as a group (e.g., power‐sharing, language and communication, authorship policy), as well as to hear from the safe space sites about their local models and specific operational and locational characteristics. The types of outcome measures and data collection methods that might be used for the evaluation were also tentatively discussed. These were explored further in a workshop held with staff from the service provider tasked with managing three of the six safe space sites.

### 3.5 Co‐designed materials and ethics challenges

Following this early work, the research team designed an online survey for site partners to provide input on outcome measures for safe space guests and staff, as well as for the health system. A range of data collection methods and tools including short and long surveys, interviews, focus groups, administrative data and arts‐based methods were included, with respondents asked to indicate their interest in using these at their site. Survey results showed a level of consistency with those presented in the funding proposal regarding health system outcomes such as sustainability, reduced emergency department presentations, and diversion from the emergency department. However, additional outcome measures for safe space guests and staff were suggested including safety, feeling heard, professional support and development and perceived value alignment between peer support work and organisational culture. Results indicated general agreement on the use of brief surveys for guests designed as short, unobtrusive tools that could be implemented as part of routine activities (entry/exit survey, evaluation wheel and graffiti wall). These tools focus on the core outcomes for the project: distress, service experience and diversion from the emergency department. The evaluation wheel also collects information on additional suggested outcomes, including feeling safe and heard. Site staff were most uncertain about implementing the guest journeys method and the arts‐based method—body‐mapping (for a description of co‐designed methods see Banfield et al.^18^). These were the most intensive and time‐consuming methods, and uncertainty about their use reflected stakeholder concerns about the discomfort they might cause to guests experiencing distress, as well as their capacity to be implemented within routine practice. However, the research team recognised that guest journeys and arts‐based methods would provide rich insights in a research space that may yield low numbers of survey responses, and one where qualitative research was highly valued. The core project team, including the stakeholder representatives, therefore chose to keep these methods in the study, to be implemented later as described below.

Based on this initial co‐design work, the research team developed a research protocol for submission to the relevant Human Research Ethics Committee (HREC) outlining the proposed project, how it would be run, and identifying any ethical risks it may pose to participants and how they would be managed. With further co‐design activities planned to ensure data collection tools were suitably user‐friendly and context‐sensitive, we adopted a staged approach to project implementation. This would allow the research team to work closely with individual sites to manage the implementation of data collection from more basic, routine procedures through to more complicated, in‐depth ones. At this juncture, the primary challenge for the research team was balancing the requirements of the HREC for explicit project detail for the purpose of determining ethical acceptability, with the need for flexibility and adaptability to accommodate ongoing input and development from co‐design partners. To this end, data collection tools submitted to the HREC for approval contained question sets that illustrated the most intrusive areas of questioning and outcomes of interest, with banks of questions from which the final set(s) were to be chosen. This allowed for the subsequent modification of approved tools after further co‐design. These were then submitted to the HREC for final approval before the commencement of data collection. As the question banks had already received approval, and there were no material differences in the ethical risks posed by the final co‐designed data collection materials, it was hoped that these could be approved out‐of‐session. However, the ethics office insisted that the materials be re‐reviewed by the full committee, delaying data collection by a further two months. This highlighted how many HREC procedures are not sufficiently equipped to accommodate robust co‐design that purposely aims to improve research merit, integrity and beneficence.

### 3.6 Key issues emerging from co‐design

One of the most pressing issues raised during co‐design was how to balance the needs for data to evaluate guest outcomes with guests' needs for care and support. Peer support workers and lived experience representatives raised concerns about the potentially onerous, intrusive and clinical nature of certain lines of questioning around suicidality and distress. Measurement of guests' distress and suicidality was an aspect of the clinical approach they had hoped to avoid within safe spaces. Although most safe space sites have allowed the measurement of a guest's distress levels using a simple scale upon entry and exit, they do so cautiously and are careful not to pressure people into engaging if they do not wish to. As well as worries over the potential of this line of questioning to cause distress or to burden those with already low mood or energy, some were concerned that the longer survey posed an additional barrier to those accessing the service. In particular, peer support workers were apprehensive about asking people to complete the survey during their visit. They thought that allowing people to complete the survey in a time and place where they felt comfortable was important. Once it was explained that the survey was opt‐in and that it would be conducted in the days or weeks following a guest's visit, peer support workers were more receptive to its use, and suggested points in their usual follow‐up procedures in which it could be implemented.

In developing the longer survey, the research team anticipated these concerns and sought to tackle them in different ways. First, the use of clinically‐worded or ‐oriented measures was minimal. Instead, questions address outcomes relevant to guests, staff and the wider system, and aim to generate data beneficial to all partners and stakeholders. Second, all questions about suicide and distress are optional, with the design enabling respondents to skip any questions or the entire section. This puts the choice of whether to respond to this line of questioning in the hands of participants, enabling full voluntary consent without forcing abandonment of participation to avoid sensitive areas. Third, co‐implementation of data collection with safe space staff has been designed to foster discussion of survey content and the risks and benefits of participation with potential research participants. Our previous work in this area suggests that people who feel well‐informed are more comfortable taking part in research and providing an honest assessment of their experiences.

Further co‐design workshops and individual meetings with peer support workers and lived experience representatives resulted in feedback on the use and meaning of certain words within the data collection materials that were polarising to people with lived experience of emotional distress and/or suicide; for example, terms such as ‘recovery’ and ‘hope’. In addition to wording, suggestions were also provided on improving the visual design of questionnaires so that graphic elements could be better utilised to improve the survey experience for participants. The research team responded to these suggestions by enabling some novel question types within the online survey software, such as ‘heat maps’ for the evaluation wheel, enabling participants to touch an area of the wheel to indicate their response instead of using a rating scale. A more inclusive approach to the wording of demographic questions was also adopted to incorporate intersectionality, and additional questions were added relating to the needs of families/carers who may present to safe spaces alone or with the person they are supporting. Finally, input from peer support workers helped to ensure that additional outcome measures of importance to them were included in the final data collection tools for staff. These included questions on how lived experience was incorporated into their work, how boundaries were managed with guests, and how the work impacted upon staff members and their wellbeing.

## 4 DISCUSSION

Collective leadership, power sharing, safety and trust are essential precursors to effective co‐creation research.^20^ Leadership competencies that nurture appropriate values, behaviours and team culture are therefore important for establishing and maintaining supportive environments where stakeholders are provided equal opportunity to influence co‐creation.^21^ Ethical issues can and do arise during co‐ideation and co‐design stages, and before the process of formal ethical review, and it is important to be mindful of these potential challenges.^22^ The principal investigator's (MB) skills and experience leading previous co‐creation projects ensured ways of working together were ethically sound and consistent with good practice during these first two stages. This included allocating sufficient time for co‐ideation and co‐design activities, inclusive communication, avoiding tokenism, providing fairness of opportunity, and valuing, acknowledging and rewarding stakeholder involvement.^22^ In the absence of previous skills and experience, numerous toolkits exist to assist researchers and organisations to undertake co‐creation research.^23^, ^24^ Informed by both local experience and international evidence, these tools can be readily applied to a variety of local contexts to assist researchers with co‐creation processes. For those new to co‐creation, explicit guidance can be helpful. However, the highly relational nature of co‐creation means that it is often messier than any ‘how to’ manuals suggest, encompassing a range of activities that continue to evolve as relationships develop and decisions are made.^25^ Any sustainable outcome is therefore dependent on interpersonal relationships and the extent to which stakeholders, including researchers, establish and maintain genuine partnerships with each other.^20^, ^25^

This leads us to reflect on whether our methods for facilitating and supporting stakeholder involvement enabled meaningful and empowered participation in co‐ideation and co‐design processes. While the project employed inclusive, representative and redistributive processes of engagement including recruitment of designated lived experience researchers and the involvement of academics, health and community service managers, policy makers, lived experience organisations and advocates, including carers, each brings with them different abilities, statuses and interests.^26^ Rather than aiming for completeness and representativeness of participation on every task, the model of collective leadership within the project has sought to allocate and distribute ‘leadership power to wherever expertise, capability and motivation sit’.^21^ The high levels of engagement in which diverse stakeholders have worked collectively to agree upon core outcomes, consolidate research methods and develop and test data collection tools provides some evidence of meaningful participation. This does not obviate the need for evaluation of the project's collaborative processes, complexity of social relations and fields of power in which equitable and meaningful participation reside.^27^ To this end, we aim to measure the structures and processes of collaboration within the project using the Collaborative Health Assessment Tool.^28^

The social relations of research production that provide the structure within which co‐creation research is undertaken warrant closer analysis, especially given the claims made by Pearce et al.^11^ that any new knowledge generated by co‐creation research ‘must derive from rigorous research methods’. Mounting disillusionment with traditional approaches to suicide research in recent decades has resulted in a growing emphasis on lived experience as a means of generating research with more meaningful and practical outcomes for those impacted by or experiencing emotional distress and/or suicidal crisis. This approach to research has been termed ‘emancipatory’ in that it is directly linked to people's ongoing struggle and demand for change through transformation of the social relations of research production.^29^ Central to this is ‘a recognition of and confrontation with power’, with the interests and needs of those being researched prioritised above those of researchers.^2^

However, Pearce et al.^11^ do not address the ethical and political aspects of co‐creation, especially as they relate to the redistribution of power among stakeholders and calls for more contextualised forms of research within suicide research and prevention.^27^, ^30^ Indeed, we read their definition and operationalisation of rigour as one that employs experimental or quasi experimental design (e.g., randomised controlled trial; step‐wedge design; multiple base design) as having a strong quantitative bias toward research that is systematised, uniform, replicable and ‘value‐free’.^31^ This narrow view of what constitutes rigour provides a poor guide for evaluating those research methods that seek to incorporate flexibility, context, engagement and reflection.^31^ Such a definition of rigour may also serve as a strategy by which some researchers seek to maintain power, credibility and the primacy of their knowledge within the co‐creation process, especially in relation to consumers, carers, or those with lived experience.^32^ While helpful as a translational model for conceptualising the co‐creation of new knowledge alongside the design and delivery of health services, Pearce et al.'s^11^ framework would appear to discount co‐created knowledge that seeks to blend research, experiential (professional, consumer, carer), and contextual knowledge to create more implementable knowledge; what Langley et al.^33^ refer to as ‘collective making’. Such knowledge is especially relevant to research, health systems, consumer and carer partnerships that view the knowledge brought by each person as legitimate, and that encourages collective ownership of outputs.^33^

Co‐creation is messy and complex with much of the work invisible and dependent on relationships and shared values.^26^ Numerous frameworks for supporting co‐created research exist from positivist to complex, context sensitive and person‐centred models.^1^, ^33^ While it is useful to think about what is involved in co‐creation, it is difficult to pin down these processes. In our experience with the Co‐Creating Safe Spaces project, the boundaries between collaborative processes are porous and do not neatly partition across categories. Rather, co‐creation activities must be dynamic to make best use of feedback, and to ensure that stakeholders' views remain relevant and can continue to shape outputs. While Pearce et al's^11^ framework introduces a useful structure for conceptualising and operationalising the collaborative process, ultimately these categorisations are a theoretical imposition upon work that is iterative, fluid and relational. Negotiating the tension between an ‘academic ideal’ and local, contextual and solutions‐focused approaches is, we contend, a defining feature of co‐creation research practice.^1^

## 5 NEXT DIRECTIONS

Following approval of our amended data collection tools by the HREC, we have commenced moving into the co‐implementation and co‐evaluation stages of the project. As the implementation of the safe spaces continues and staff respond to guest feedback and other emergent challenges, including incorporating the processes of data collection within their daily practice, we expect a similar fluidity and movement back and forth between different collaborative processes. To help us better understand the dynamic nature of our collaboration, we propose to evaluate it using an augmented Collaborative Health Assessment Tool that will measure it according to four structure dimensions (i.e. shared goal, shared resources, shared authority and shared accountability) and four process dimensions (i.e. whole‐system engagement, communication flows, adaptive capacity and holding/authorising environment).^28^ This will help us to identify which dimensions of collaboration need to be improved upon.

## 6 CONCLUSION

To support meaningful participation, incorporate the interests of diverse stakeholders, and comply with multiple bureaucratic processes across the entirety of the research process, co‐created research is necessarily iterative, fluid and deeply relational. The boundaries between collaborative processes that make up this work, therefore, are not easily drawn. Although important, the focus on definitional issues and the application of consistent terminology within some theoretical frameworks may mask important issues of representation, engagement, the distribution of power and evidence hierarchies in health research practice. Drawing on our experiences of designing and coordinating the Co‐Creating Safe Spaces project, we suggest that the social relations of research production that provide the structures within which all co‐created knowledge is generated are more important drivers of effective knowledge mobilisation and implementation, especially within the contested field of suicide prevention. Closer attention to these social relations is needed to ensure co‐creation activities align with the foundational principles of co‐creation: power‐sharing, equity and the transformation of health and social systems.

## AUTHOR CONTRIBUTIONS

**Scott J. Fitzpatrick**: Writing—original draft, review and editing; conceptualisation; project administration; supervision. **Heather Lamb**: Writing—original draft, review and editing; conceptualisation; funding acquisition. **Erin Stewart**: Writing—original draft, review and editing; conceptualisation. **Amelia Gulliver**: Writing, reviewing and editing; conceptualisation; funding acquisition. **Alyssa R. Morse**: Writing, reviewing, and editing; funding acquisition. **Melanie Giugni**: Writing, reviewing and editing; project administration. **Michelle Banfield**: Writing—original draft, review and editing; conceptualisation; project administration; supervision; funding acquisition.

## CONFLICT OF INTEREST STATEMENT

The authors declare no conflict of interest.

## Data Availability

Data sharing is not applicable to this article as no data sets were generated or analysed during the preparation of this article.
